# Effects of Resistance Exercise with or without Whey Protein Supplementation on Ocular Changes after a 21-Day Head-Down Bed Rest

**DOI:** 10.3390/life11080741

**Published:** 2021-07-26

**Authors:** Marc Kermorgant, Sirine Hammoud, Laurence Mahieu, Thomas Geeraerts, Arnaud Beck, Marie-Pierre Bareille, Vincent Soler, Anne Pavy-Le Traon, Jean-Claude Quintyn

**Affiliations:** 1 INSERM DR Midi-Pyrénées Limousin, Institute of Cardiovascular and Metabolic Diseases (I2MC) UMR1297, University Hospital of Toulouse, 31432 Toulouse, France; marc.kermorgant@gmail.com; 2 Department of Neurology, University Hospital of Toulouse, 31059 Toulouse, France; 3 Department of Ophthalmology, Glaucoma Institute, Saint Joseph Hospital, 75014 Paris, France; sirine.hammoud@gmail.com; 4 Department of Ophthalmology, University Hospital of Toulouse, 31059 Toulouse, France; mahieu.l@chu-toulouse.fr (L.M.); soler.v@chu-toulouse.fr (V.S.); 5 Department of Anesthesiology and Intensive Care, University Hospital of Toulouse, 31059 Toulouse, France; geeraerts.t@chu-toulouse.fr; 6 Institute for Space Medicine and Physiology (MEDES), 31405 Toulouse, France; arnaud.beck@medes.fr (A.B.); marie-pierre.bareille@medes.fr (M.-P.B.); 7 Department of Ophthalmology, Unicaen, University Hospital of Caen, 14033 Caen, France

**Keywords:** optical coherence tomography, intraocular pressure, head-down bed rest, microgravity

## Abstract

Neuro-ophthalmological changes have been reported after prolonged exposure to microgravity; however, the pathophysiology remains unclear. The objectives of the present study were twofold: (1) to assess the neuro-ophthalmological impact of 21 days of head-down bed rest (HDBR) and (2) to determine the effects of resistance vibration exercise (RVE) alone or combined with nutritional supplementation (NeX). In this case, 12 healthy male subjects completed three interventions of a 21-day HDBR: a control condition without countermeasure (CON), a condition with resistance vibration exercise (RVE) comprising of squats, single leg heel and bilateral heel raises and a condition using also RVE associated with nutritional supplementation (NeX). Intraocular pressure (IOP) was assessed by applanation tonometry. Retinal nerve fiber layer thickness (RNFLT) was assessed with spectral-domain optical coherence tomography, before HDBR and between Day 2 and Day 4 after each session of HDBR. In CON condition, IOP was preserved; while in RVE and NeX conditions, IOP was increased. In CON condition, RNFLT was preserved after HDBR. RVE and NeX conditions did not have significant effects on RNFLT. This study showed that a 3-week HDBR did not induce significant ophthalmological changes. However, RVE induced an elevation in IOP after HDBR. Nutritional supplementation did not reduce or exacerbate the side effects of RVE.

## 1. Introduction

Changes in astronauts’ visual function and ocular structure have been reported after long duration spaceflights defined by NASA as Spaceflight-Associated Neuro-ocular Syndrome (SANS) [1]. Some astronauts developed some level of optic disc edema after long-duration spaceflight [2]; with only partial recovery one year after landing [1]. Indeed, some crewmembers who underwent the environment on the International Space Station during several months, exhibited a large panel of ophthalmologic issues including enlargement in optic nerve sheath diameter, a rise in intraocular pressure (IOP), papilledema, increase in circumpapillary retinal nerve fiber layer thickness (RNFLT), optic disc edema, choroidal folds, hyperopic shift, globe flattening, etc. All these phenomena may reflect a rise in intracranial pressure (ICP). Another hypothesis explaining SANS was a compartmentalization of cerebrospinal fluid within the orbital optic nerve sheath [2,3,4,5,6,7]. Thus, the description of these visual changes is questioning the medical space community on safety aspects of long duration spaceflights.

Countermeasures (such as muscular exercise, dietary regimen, thigh cuffs, etc.) have been tested to counteract the harmful effects of microgravity. However, most of these countermeasures provides partial protection. During spaceflight, resistance exercise and dietary regimen are employed in order to lessen bone loss or muscle wasting [8,9]; however, they were also considered as risk factors contributing to an increased ICP during spaceflight [2,10]. Very little data is available on the impact of these countermeasures on ocular changes (and consequently on ICP) in microgravity. However, it was previously demonstrated that resistance exercise might induce a rise in IOP [11,12,13]. NASA was also recommended a low sodium consumption to avoid developing visual outcomes [12,14].

Ground-based model of microgravity are primordial for determining the impact of weightlessness on crewmembers’ health. Head-down bed rest (HDBR) is one of the most popular analogues to microgravity. The subject remains in supine position on a bed at −6 degrees for very variable periods (from days to months). This model reproduces several physiological changes encountered during spaceflight such as, immobilization, inactivity, cardiovascular deconditioning, alteration in vestibular function, etc. [11]. The purpose of the present study was twofold: (1) to assess the neuro-ocular modifications induced by a 21-day HDBR, and (2) to determine whether resistance vibration exercise (RVE) alone or combined with nutritional supplementation (NeX) may alter these HDBR-induced ophthalmological adaptations. We hypothesized that HDBR will provoke thoraco-cephalic fluid resulting in an increase in ICP as reflected by ophthalmological changes. We also made the assumption that RVE and NeX countermeasures could strengthen the effects of HDBR on ICP.

## 2. Materials and Methods

### 2.1. Subjects

This study (registered number: 2012-A00337-36) was carried out with the recommendations of the Ethics Committee (CPP Sud-Ouest Outre-Mer I). The protocol was approved by the French Health Authorities. All subjects gave written informed consent in accordance with the Declaration of Helsinki. The study was performed by the Institute for Space Medicine and Physiology (MEDES-IMPS) in Toulouse, France and supported by the French Spatial Agency [Centre National d’Etudes Spatiales (CNES)]. 12 healthy men (at selection: 34 ± 7 years; 176 ± 7 cm; 70 ± 7 kg) were included in the study.

The inclusion criteria were healthy male volunteer, age: 20–45 years, height: 158–190 cm, BMI: 20–26 kg/m^2^, no family nor personal past record of acute or chronic diseases, no psychological disturbances, fitness assessment for <35 years: 35 mL/min/kg < VO_2max_ < 60 mL/min/kg and >35 years: 30 mL/min/kg < VO_2max_ < 60 mL/min/kg, no orthopedic, musculoskeletal or cardiovascular disorders, and no history of regular smoking, no alcohol, no drug dependence and no medical treatment.

The non-inclusion criteria were orthostatic intolerance, arterial hypertension and cardiac rhythm disorders. Glaucoma, optic nerve known pathology or eye injury that could interfere with the results and their interpretation. Chronic back pains, vertebral fracture, scoliosis or herniated disc, history of knee problems or joint surgery/broken leg. History of hiatus hernia or gastro-esophageal reflux, thyroid dysfunction, renal stones, diabetes and migraines. Past records of thrombophlebitis, family history of thrombosis or positive response in thrombosis screening procedure. Abnormal result for lower limbs in Doppler ultrasound. Bone mineral density: *T*-score ≤ −1.5, osteosynthesis material, presence of metallic implants. Poor tolerance to blood sampling and having donated blood (more than 8 mL/kg) in a period of 8 weeks or less before the start of the experiment.

### 2.2. General Description

**Figure 1:**
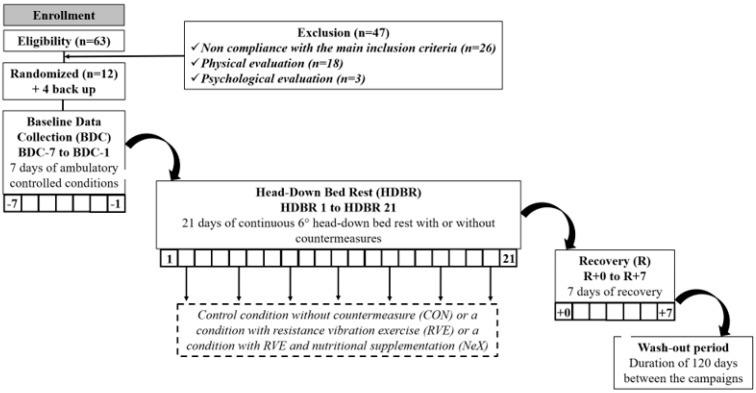
Flow chart of the study.

Reproducing the space environment by abolishing the longitudinal gravitational stress, the HDBR consists in a bed-rest in a 6° head-down tilt position. The ophthalmological assessments performed were part of the medical follow-up. During the HDBR period, subjects were randomly divided into three groups according to the countermeasures tested: a control group (no countermeasure), a group performing resistance exercise with vibrations and a group performing resistance exercise with vibrations associated with nutritional countermeasures. This study included 3 sessions of 35 days of hospitalization, each with a 21-day HDBR session. Each session was separated from the next one by a period of 3 months. A flow chart of the study is represented Figure 1.

**Figure 2:**
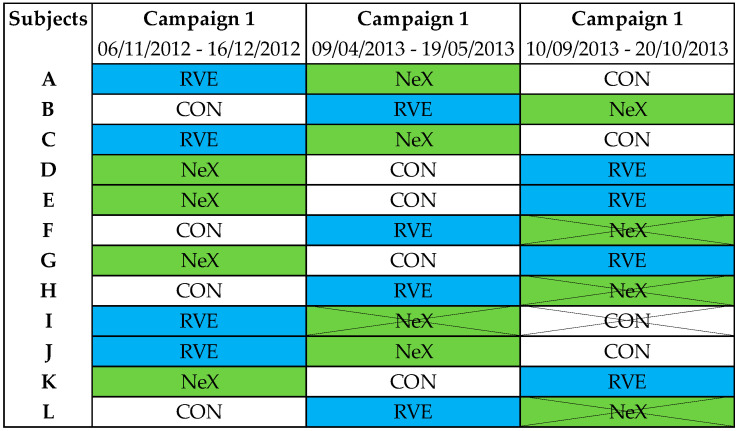
Allocations for each subject. “CON” denotes control condition, “NeX” denotes resistance vibration exercise with nutritional supplementation, “RVE” denotes resistance vibration exercise condition.

The order of the three interventions during HDBR was randomized: none (CON), resistance vibration exercise (RVE) alone or combined with nutritional supplementation (NeX) with the following sequences (CON/RVE/NeX or RVE/NeX/CON or RVE/CON/NeX). Four subjects withdrew from the study, one during the second campaign and three others during the third campaign. Ophthalmologic data were completed in eight subjects; therefore, we performed a per-protocol analysis (Figure 2).

### 2.3. Countermeasures Description

#### 2.3.1. Resistance Vibration Protocol

During each session, the sequence was performed as follows: (a) warm up consisted to bilateral squats from 10° to 90° knee angle during 8 s (4 down, 4 up) controlled by metronome with 8 repetitions, load: 50% of the one repetition maximum (1-RM), amplitude: 8 mm, vibration frequency: 24 Hz. (b) squatting exercise was realized by bilateral squats from 10° to 90° knee angle during 8 s (4 down, 4 up) controlled by metronome with 10 repetitions, load at study start: 75% of the 1-RM, progression: 5% load increase when more than 10 repetitions were possible, 5% load decrease when 6 or fewer repetitions were possible, amplitude: 8 mm, vibration frequency: 24 Hz. (c) single leg heel raises were carried out from maximal dorsiflexion to maximal plantar flexion as fast as possible until exhaustion, 1.3 times body weight (bw), progression: 5% load increase when more than 50 s were possible, 5% load decrease when 30 s or less were possible, amplitude: 8 mm, vibration frequency: 26 Hz. (d) bilateral heel raises were performed from maximal dorsiflexion to maximal plantar flexion as fast as possible until exhaustion, 1.8 times bw, progression: 5% load increase when more than 55 s were possible, 5% load decrease when 40 s or less were possible; amplitude: 8 mm, vibration frequency: 26 Hz.

#### 2.3.2. Nutritional Supplementation

An isocaloric supplementation of whey protein (0.6 g/kg bw/day) was given to the volunteers of the nutritional and exercise intervention sequence. The total protein intake was 1.8 g/kg bw/day. The schedule of the protein supplementation was the following: (1) on days without exercise applied in equal amounts with main meal and (2) on days with exercise, half of the daily amount in a timeframe of 30 min after exercise and the other half equally distributed with main meals. The product was Diaprotein^®^, a powder supplied by Nephrologische Präparate Dr. Volker Steudle (Linden, Germany). The composition was as follows: Diaprotein^®^ 100 g Powder, calorie intake 1573 kJ (370 kcal), proteins 90 g, fat 0.2 g, lactose 2.5 g, sodium <300 mg, potassium <650 mg, calcium <400 mg, phosphorus <250 mg, relation phosphorus/protein <3 mg/g. Since whey protein added a certain acid load to the diet, supplementation of 90 mmol potassium bicarbonate per day, applied in six portions (with main meals) was given to compensate for that. Effervescent tablets of potassium bicarbonate were provided by Krüger GmbH and Co. (Bergisch Gladbach, Germany). The detailed profile of the supplement has been described previously [12].

### 2.4. Intraocular Pressure Measures

A topical local anesthetic solution (Tetracaïne, 1% per ophthalmic drops) was instilled in each eye just prior to evaluation. IOP was performed at specific times between 2 p.m. and 4 p.m. The IOP measurements were performed at rest in supine position, in both eyes, once a day, with a Perkins tonometer (Perkins MK2, Haag Streit, Luneau, Chartres, France) 4 days before (BDC-4) and 1 day after (R + 1) HDBR. The final measure corresponds to the average of these two measures.

### 2.5. Optical Coherence Tomography Measures

The measurements were performed at rest in seated position before HDBR (BDC) and between Day 2 and Day 4 after each session of HDBR (R), with automatic follow-up scans placement, by trained ophthalmologists. OCT was performed to quantify the RNFLT, as a reflection of a disc swelling. Spectral domain OCT images were obtained with the Spectralis OCT (Heidelberg Engineering software version 5.1.3.0, GmbH, Heidelberg, Germany). The quality for each measurement was determined by a quality index provided by the OCT device. Measurements not fulfilling this condition were automatically eliminated and repeated. Optic nerve head thickness was divided in four quadrants: temporal, nasal, superior and inferior. If the quality index of a measurement provided by OCT device was not sufficient, each measurement was eliminated and repeated. Right and left eyes were assessed. The final measure corresponds to the average of the two measures. All OCT measurements were validated by experts (Laurence Mahieu, Vincent Soler and Jean-Claude Quintyn) blinded to the condition.

### 2.6. Eye Examination

An examination with slit lamp was also performed for each patient.

### 2.7. Statistical Analysis

Data were expressed as mean ± SD. We first checked whether data passed Shapiro–Wilk normality test. A Bartlett’s test was applied in order to verify the homoscedasticity of variances (*p* > 0.05). Two-way ANOVA with repeated measures with a Geisser–Greenhouse correction was used with Sidak’s multiple comparisons test. Subjects were entered as random factors and condition and time were included as fixed factors. All statistical analyses were performed with GraphPrism 9. Differences were considered as statistically significant when *p* < 0.05.

## 3. Results

### 3.1. Eye Examination

At the back of the eye, no lesion was seen, no papilledema, no hemorrhage before and after HDBR. We did not ever observe venous pulsations of the optic nerve.

### 3.2. Intraocular Pressure Measures

**Figure 3:**
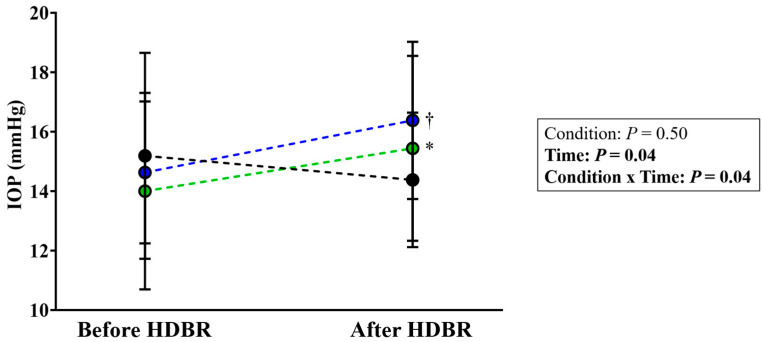
Intraocular pressure (IOP) before (BDC-4) and after (R + 1) head-down bed rest. Black dots denotes control condition, blue dots denotes resistance vibration exercise (RVE) condition, green dots denotes resistance vibration exercise with nutritional supplementation (NeX) condition.

In CON condition, compared to BDC-4, IOP was not significantly modified during R + 1 (15.2 ± 3.5 mmHg vs. 14.4 ± 2.3 mmHg; *p* = 0.75). However, IOP was significantly increased both in RVE (14.6 ± 2.4 mmHg vs. 16.4 ± 2.6 mmHg; *p* = 0.04) and NeX (14.0 ± 3.3 mmHg vs. 15.4 ± 3.1 mmHg; *p* = 0.03) conditions during R + 1 (Figure 3).

### 3.3. Optical Coherence Tomography Measures

There were condition effects for RNFLT in temporal quadrant (*p* = 0.04). However, Sidak post hoc test did not reveal any significant differences between groups. We also noticed non-significant interaction effects for RNFLT in nasal quadrant. We did not observe any significant modifications in RNFLT, neither in the superior quadrant nor in the inferior quadrant. The average RNFLT was also preserved (Table 1).

## 4. Discussion

**Intraocular pressure preserved after head-down bed rest, but impaired with resistance exercise with or without nutritional supplementation.**

Few data are available during spaceflight. However, Draeger et al. [13] showed a slight but significant increase in IOP (~5 mmHg) after exposure to microgravity in a 8-day German Spacelab. The same authors found similar trends in a 10-day Spacelab D2 mission. Indeed, only 15 min after entering in microgravity, IOP was increased (~12–13 mmHg), but returned to ground-based values on the day 4 [14]. Stenger et al. [15] gathered IOP data in 11 astronauts from 6 shuttle missions and described preserved post-flight IOP values compared to pre-flight IOP values. Irrespective of the duration of the spaceflight and an immediate rise in IOP after entering microgravity; IOP values seem to normalize, although cranial venous fluid shift were maintained [16]. Most of the results performed during HDBR are contradictory since some studies showed either a reduced or an increased IOP. In our study, we did not observe any significant changes in IOP after 21 days of HDBR. Chiquet et al. [17] reported in a 7-day HDBR performed in young healthy female volunteers, a drop in IOP associated with hypovolemia related to cephalad fluid shifts followed by an increase in IOP after HDBR. The authors suggested that these ocular changes were mainly due to ocular dehydration or to systemic cardiovascular and hormonal variations during HDBR. The measurement of IOP in the same study is important since the pressure gradient between ICP (which would increase) and IOP (which would remain stable or decrease) may be one of the factors favoring optic nerve edema [18]. A case report performed in a 25-year-old Caucasian man who underwent a 30-day HDBR displayed the same trend with a diminution in IOP. This phenomenon could contribute to a decreased translaminar pressure [19]. In contrast, Taibbi et al. [20] showed that 14- and 70-day HDBR provoked an increase in IOP (respectively, +1.42 and +1.79 mmHg) but returned to baseline values after HDBR. The magnitude of the increase observed during HDBR was not associated with the campaign durations.

Several studies showed that even a short-duration spaceflight induced musculoskeletal injuries and cardiovascular deconditioning [21,22]. By this way, repeated resistance exercise countermeasures become essential for crewmembers’ health during mission on the International Space Station [23,24]. To date, neither IOP nor ICP measurements were performed during or after resistance exercise onboard the International Space Station [25]. Furthermore, it has been previously suggested that repeated performance of heavy-load resistance exercise has been proposed as a contributing factor to an elevated ICP and ocular outcomes during spaceflight [26]. Several studies have attempted to determine the impact of resistance exercise on IOP. However, data are conflicting. Dickerman et al. [27] depicted in 11 athletes a massive increase in IOP with the Valsalva maneuver during heavy resistance exercise with maximal intensity (+15 mmHg). Vieira et al. [8] observed similar trends where 30 healthy male volunteers have undergone 4 repetitions of bench press with and without breath holding during the last repetition. They observed a rise in IOP during a bench press session either with (+4.3 mmHg) or without (+2.2 mmHg) breath holding. In contrast, Avunduk et al. [28] described a reduction in IOP during both isometric and isokinetic exercises of the lower body, with a greater reduction during isokinetic exercise (−7 mmHg) than isometric exercise (−4 mmHg). Chromiak et al. [29] found a reduction in IOP in 30 healthy subjects (15 males and 15 females) after 3 sets of 10 repetitions of the chest press and leg press exercises with 70% 1-RM. Rüfer et al. [30] did not observe any significant changes in IOP during resistance exercise lower limb, while IOP was slightly increased during resistance exercise upper limb (20 repetitions with 65% W_max_ at the butterfly machine). Nevertheless, irrespective of the type of the exercise, IOP returned to baseline values. It is noteworthy that the majority of exercise studies have been performed in seated position; however, only very few of them determined the impact of resistance exercise on IOP during HDBR position. One study demonstrated a small decrease in IOP (−1.61 mmHg) after a short session of weightlifting in 25 healthy volunteers in supine position with 85% top load for 8 repetitions [31]. Taibbi et al. [32] studied the effects of a 70-day HDBR on ophthalmic changes with some specific countermeasures such as integrated resistance and aerobic training in 6 controls and 9 exercisers. The authors declared that both controls and exercisers exhibited a rise in IOP during HDBR (respectively, +1.38 mmHg and +1.63 mmHg), measured with iCare IOP. They also concluded that no difference was observed between these two groups. However, the authors found a greater magnitude of change in Goldmann applanation tonometry IOP in exercisers compared to controls after HDBR (+1.14 mmHg vs. −0.47 mmHg). Consistently in our study, we found that resistance exercise induced a slight but significant elevation in IOP (+1.8 mmHg) after HDBR. The major differences observed in IOP variations reported in the literature are mainly due to the fact that most exercise studies have been performed in seated position. Furthermore, as suggested previously by Marshall-Bowman [26], heavy resistance exercise combined with the effects of microgravity are prone to be involved in the development of ophthalmological outcomes.

Zwart et al. [33] showed that high-protein diet may induce a low-grade metabolic acidosis. Thus in our study, an additional supplementation with potassium bicarbonate was applied in order to counteract the potential low-grade metabolic acidosis, as previously detailed in some studies [34,35]. Blottner et al. [36] has previously attempted to determine whether whey protein plus potassium bicarbonate supplement may impact on disused muscle after HDBR. Even though they found an attenuated disuse-induced reductions in muscle fiber oxidative capacity after HDBR [37]; whey protein with potassium bicarbonate supplement failed to prevent skeletal muscle atrophy [36]. However, little is known about the impact of potassium and sodium supplementation on IOP. It has been shown in 15 patients with glaucoma that administration of potassium chloride (58 mmol) did not lower IOP [38]. It has also been proven that high sodium levels produced an osmotic shift of body fluid from the interstitial to the intravascular compartment leading to venous congestion [9]. Consequently, in our study it is unlikely that low supplementation with potassium bicarbonate (90 mmol) may affect IOP. Furthermore, Stenger et al. [15] suggested that the association between microgravity-induced cranial fluid shift and an elevated sodium intake (which may expand the extracellular fluid volume) might have deleterious synergic effects on the ICP. NASA recommended to drastically reducing the daily sodium intake, below 3 g per day [10] to prevent visual outcomes [9]. A previous study demonstrated the strong relationship between elevated ICP and sodium and water retention. Indeed, 77% patients with idiopathic intracranial hypertension had peripheral edema and 80% had orthostatic retention of sodium and water [39]. Newborg [34] has observed a total remission of papilledema in 9 patients who were initially treated for intracranial hypertension. In their diet, inter alia, patients’ sodium intake was less than 100 mg per day. However, the authors mentioned that it was difficult to determine whether the enhancement observed was due to either weight loss or sodium and water distribution. In our study, nutritional supplementation did not enhance or worsen the side effects of resistance exercise. Indeed, it was unlikely that low sodium intake (<300 mg) had an impact on the ophthalmological changes induced by resistance exercise.

**Retinal nerve fiber layer thickness preserved after head-down bed rest, no effect of resistance exercise with or without nutritional supplementation.**

Degradation in visual acuity such as hyperopic shift or residual choroidal folds have been previously observed during spaceflight [3,40]. Mader et al. [41] showed in a 57-year-old astronaut, who underwent 2 long-durations spaceflight, ophthalmological issues such as unilateral choroidal folds and a single cotton wool spot during the 1st mission. These phenomena were exacerbated during the 2nd mission. Moreover, 29% and 60% of crewmembers presented deterioration in distant and near visual acuity after short- and long-duration spaceflights, respectively [35]. Mader et al. [42] described in a case study, an asymmetric increase in total retinal thickness determined by OCT. It is noteworthy that these changes were always observed after 1 year. Patel et al. [43] documented similar findings in 15 astronauts after long-duration spaceflight. In their study, OCT scans depicted a rise in total retinal thickness and global circumpapillary RNFLT (~20 µm) with a more pronounced increase in the inferior RNFL quadrant. Macias et al. [3] noticed the same trends. Indeed, the authors noted an increase in global total retinal thickness in 11 astronauts after long-duration missions to the International Space Station (4 to 6 months). The same authors found a very high interindividual variability in 11 crewmembers. Only 2 of them had an increase in total retinal thickness. Moreover, choroidal folds and optic disc edema lasted over 1 year. Even though no optic disc edema was discerned, an increase in peripapillary retinal thickness (~19.4 µm), measured by spectralis OCT, was found in a healthy 25-year old male astronaut after a 30-day HDBR [19]. It appeared that a 30-day strict HDBR would develop more pronounced ocular changes than a “classical” 70-day HDBR, with cases of papilledema and an increase in peripapillary total retinal thickness [44]. Surprisingly, they also observed a greater peripapillary total retinal thickness in healthy subjects after 30-day strict HDBR vs. astronauts during ~30 days of spaceflight [45]. The authors suggested that subjects exposed to strict HDBR had developed a moderate ICP but greater than that observed in astronauts during similar duration of spaceflight. Time duration appears to play a key role on the development of the amount of optic disc swelling. Indeed, spectralis OCT revealed that 70-day HDBR (6 subjects) provoked greater peripapillary retinal thickening compared to 14-day HDBR (16 subjects) [20]. Glaucoma is an optic neuropathy in which electrophysiological response is altered. Thus, it would be interesting to extend subsequent studies to electrophysiological methods. Indeed, Bessler et al. [46] used silent substitution stimulation to detect objectively primary open-angle glaucoma in a large cohort of patients. Moreover, the impact of the hydrostatic effects on retinal vessel diameter should not be overlooked. It has been previously demonstrated in 15 young healthy subjects that Mayer waves led to significant variations in retinal vessel diameter [47]. Moreover, Louwies et al. [48] showed that hydrostatic effect might impact on central retinal arteriolar equivalent during a 21-day hypoxic bed rest performed in 11 subjects.

Only few studies determined the impact of resistance exercise on RNFLT. Laurie et al. [45] reviewed exercise logs and findings from retrospective ophthalmological data from 34 crewmembers who spent several months on the International Space Station over 12 years. 8 of them presented optic disc edema. They mentioned that astronauts, with or without optic disc edema, spent the same amount of time to performing either aerobic or resistance exercise. A study performed in 24 healthy volunteers assessed the impact of weightlifting on ocular changes [42]. The authors described a preserved retinal thickness, ganglion cell layer and choroidal thickness after weightlifting. However, Taibbi et al. [32] also studied the effects of integrated resistance and aerobic training on circumpapillary RNFL and peripapillary retinal thicknesses after 70 days of HDBR. In this study, spectralis OCT revealed an increase in circumpapillary RNFLT (+1.33 µm) in 15 subjects (6 controls and 9 exercisers) after HDBR. They also described a peripapillary retinal thickening in controls (+9.77 μm) and exercisers (+6.65 μm), but no difference was found between these two groups. However, in our study resistance exercise did not affect significantly RNFLT after HDBR. Time duration may explain the lack of effects of resistance exercise on RNFLT.

## 5. Limitations

Some limitations must be taken into account. The study was performed with a limited number of subjects (*n* = 8) due to some withdrawals during the last campaign (*n* = 4). However, the subjects were their own controls. The frequency of exercise was only performed twice a week, which may limit the effects of the countermeasures.

## 6. Conclusions

In summary, these findings suggest that 21 days of HDBR did not induce significant ophthalmological changes. However, RVE provoked a rise in IOP. Nutritional supplementation did not enhance or worsen the side effects of RVE. In conclusion, it would be valuable to optimize the exercise in order to avoid ophthalmological outcomes. Moreover, it would be necessary to find out whether changes in retinal nerve fibers, IOP and ICP might be observed during higher frequency resistance exercises, as those conducted by astronauts during spaceflight.

## Figures and Tables

**Table 1:** Optical coherence tomography data before (BDC) and after (R) head-down bed rest.

	CON		RVE		NeX		ANOVA Table Condition, Time, Condition × Time
	*BDC*	*R*	*BDC*	*R*	*BDC*	*R*	
Average (µm)	101.4 ± 9.5	101.1 ± 8.2	100.0 ± 8.9	101.4 ± 7.6	100.7 ± 8.4	101.8 ± 8.6	*p* = 0.20, *p* = 0.18, *p* = 0.10
	(93.5–109.3)	(94.3–107.9)	(92.6–107.5)	(95.0–107.7)	(93.7–107.8)	(94.6–109.0)	
Superior (µm)	125.5 ± 15.8	126.1 ± 15.1	124.1 ± 15.3	125.1 ± 12.6	125.3 ± 13.9	126.5 ± 14.5	*p* = 0.24, *p* = 0.25, *p* = 0.86
	(112.3–138.7)	(113.5–138.7)	(111.3–136.8)	(114.6–135.6)	(113.7–136.9)	(114.3–138.6)	
Nasal (µm)	77.9 ± 13.8	76.8 ± 12.6	76.4 ± 13.8	77.5 ± 12.6	75.2 ± 12.8	77.1 ± 13.5	*p* = 0.33, *p* = 0.35, *p* = 0.06
	(66.4–89.4)	(66.2–87.3)	(64.9–88.0)	(67.0–88.0)	(64.5–85.9)	(65.7–88.4)	
Inferior (µm)	131.1 ± 16.1	130.8 ± 14.6	129.3 ± 16.5	131.4 ± 16.3	130.2 ± 16.8	130.7 ± 16.7	*p* = 0.63, *p* = 0.18, *p* = 0.16
	(117.6–144.5)	(118.6–142.9)	(115.5–143.2)	(117.8–145.1)	(116.2–144.2)	(116.7–144.6)	
Temporal (µm)	71.1 ± 8.1	70.8 ± 8.9	70.3 ± 8.6	71.4 ± 9.8	72.1 ± 8.3	73.1 ± 9.7	*p* = 0.04, *p* = 0.37, *p* = 0.27
	(64.3–77.9)	(63.4–78.3)	(63.1–77.4)	(63.2–79.5)	(65.2–79.1)	(65.0–81.2)	

“BDC” denotes baseline data collection, “CON” denotes control condition, “NeX” denotes resistance vibration exercise with nutritional supplementation, “R” denotes recovery, “RVE” denotes resistance vibration exercise condition. Data are expressed as mean ± SD. Values in parentheses represent 95% CI of the mean.

## Data Availability

All datasets generated for this study are included in the manuscript.
